# Attempting rigour and replicability in thematic analysis of qualitative research data; a case study of codebook development

**DOI:** 10.1186/s12874-019-0707-y

**Published:** 2019-03-28

**Authors:** Kate Roberts, Anthony Dowell, Jing-Bao Nie

**Affiliations:** 1 Department of Primary Health Care & General Practice, University of Otago, Wellington, New Zealand; 2 Department of Primary Health Care & General Practice, University of Otago, Wellington, New Zealand; 3 Bioethics Centre, University of Otago, Dunedin, New Zealand

**Keywords:** Thematic analysis, Rigour, Qualitative research, Codebook, Coding

## Abstract

**Background:**

Navigating the world of qualitative thematic analysis can be challenging. This is compounded by the fact that detailed descriptions of methods are often omitted from qualitative discussions. While qualitative research methodologies are now mature, there often remains a lack of fine detail in their description both at submitted peer reviewed article level and in textbooks. As one of research’s aims is to determine the relationship between knowledge and practice through the demonstration of rigour, more detailed descriptions of methods could prove useful. Rigour in quantitative research is often determined through detailed explanation allowing replication, but the ability to replicate is often not considered appropriate in qualitative research. However, a well described qualitative methodology could demonstrate and ensure the same effect.

**Methods:**

This article details the codebook development which contributed to thematic analysis of qualitative data. This analysis formed part of a mixed methods multiphase design research project, with both qualitative and quantitative inquiry and involving the convergence of data and analyses. This design consisted of three distinct phases: quantitative, qualitative and implementation phases.

**Results and conclusions:**

This article is aimed at researchers and doctoral students new to thematic analysis by describing a framework to assist their processes. The detailed description of the methods used supports attempts to utilise the thematic analysis process and to determine rigour to support the establishment of credibility. This process will assist practitioners to be confident that the knowledge and claims contained within research are transferable to their practice. The approach described within this article builds on, and enhances, current accepted models.

## Background

Navigating the world of thematic qualitative analysis can be challenging. Thematic analysis is a straightforward way of conducting hermeneutic content analysis which is from a group of analyses that are designed for non-numerical data. It is a form of pattern recognition used in content analysis whereby themes (or codes) that emerge from the data become the categories for analysis. These forms of analysis state that the material as a whole is understood by studying the parts, but the parts cannot be understood except in relation to the whole [1]. The process involves the identification of themes with relevance specific to the research focus, the research question, the research context and the theoretical framework. This approach allows data to be both described and interpreted for meaning.

In qualitative research replication of thematic analysis methods can be challenging given that many articles omit a detailed overview of qualitative process; this makes it difficult for a novice researcher to effectively mirror analysis strategies and processes and for experienced researchers to fully understand the rigour of the study. Even though descriptions of code book development exists in the literature [2, 3] there continues to be significant debate about what constitutes reliability and rigor in relation to qualitative coding [1]. In fact, the idea of demonstration of rigour and reliability is often overlooked or only briefly discussed creating difficulties for replication.

Research aims to determine the relationship between knowledge and practice through the demonstration of rigour, validity and reliability. This combination helps determine the trustworthiness of a project. This is often determined through detailed explanations of methods allowing replication and thus the application of findings, but the ability to replicate is often not considered appropriate in qualitative research. However, general consensus states that all research should be open to critique, which includes the integrity of the assumptions and conclusions reached [4]. That considered, a well described qualitative methodology utilising some components of quantitative frameworks could potentially have the same effect.

When research is aimed at informing clinical practice, determining trustworthiness is as an important step to ensure applicability and utility [5]. It is suggested that validity, one component of trustworthiness in qualitative research, can be established by investigating three main aspects: content (sampling frame and instrument development description); criterion-related (comparison and testing of the instrument and analysis tools between researchers, e.g. inter-rater or inter-coder testing); and, construct validity (appropriateness of data-led inferences to the research question using reflexive techniques) [4]. It would thus seem then that determining the validity, or ‘trustworthiness’, can be best achieved by a detailed and reflexive account of procedures and methods, allowing the readers to see how the lines of inquiry have led to particular conclusions [6].

Whilst the development of a codebook is not considered a time efficient mode for analysis [2], it enables a discussion and possibility of replication within qualitative methods utilising what could be considered a quantitative tool. It also allows reliability testing to be more easily applied. The codebook development discussed in this article formed part of a mixed methods multiphase design research project (mentioned throughout as the “case study”) with the overarching aims of identifying barriers and enhancing facilitators to communication between two health provider groups. The discussion of demonstration of codebook development will include examples from the PhD research project, and whilst a full discussion of the project is not within the scope of this article some background will assist in a grounding of the discussion.

**Fig. 1:**
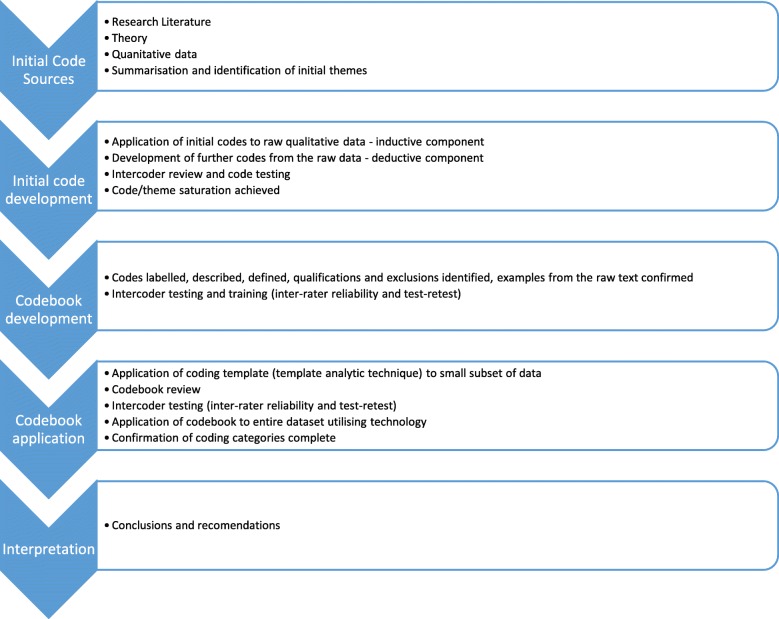
- Process of code creation and testing

This research project was a mixed methods multiphase design project, with both qualitative and quantitative inquiry and involving the convergence of data and analyses. This design consisted of three distinct phases: quantitative, qualitative and implementation phases. This project’s qualitative thematic analysis utilised the dualistic technique of inductive and deductive thematic analysis informed by the work of Fereday and Muir-Cochrane which included the development and description of an analytical codebook [7]. (See Fig. 1 for the process followed in the coding process) The deductive component involved the creation of a preliminary codebook to help guide the analysis. This was based on the research question being asked, the initial analysis of the literature, the quantitative survey undertaken as part of the project and a preliminary scan of the raw interview data [8]. Additionally, the inductive approach followed the creation of the codebook. This allowed for any unexpected themes to develop during the coding process [9]. Deductive approaches are based on the assumption that there are ‘laws’ or principles that can be applied to the phenomenon. Insight was thus derived from the application of the deductive model to the set of information and searching for consistencies and anomalies. Conversely, inductive approaches searched for patterns from the ‘facts’ or raw data [9]. This allowed for any unexpected themes with the potential to provide further useful analysis of the data to develop during the coding process. Combining these approaches allowed the development of patterns from the unknown parts that may fall outside the predictive codes of deductive reasoning and allowed for a more complete analysis.

This combined inductive/deductive approach fits well with both a mixed methods style of methodology and a pragmatic epistemology underpinning, whereby the methods are chosen by the researcher to be best able to answer the research questions. A critical realism ontological approach also meant that while the deductive approach provided an initial sound grounding, the creator of the codebook needed to include an inductive process to allow the reality of others to be clearly represented in the data analysis.

The goal of this article therefore is to highlight the difficulties of the demonstration of rigour in qualitative thematic analysis. It does this by investigating the assumption which states that replicability is not seen as necessary in qualitative research. It then continues this conversation by showing the process of a codebook development and its use as a means of analysing interview data, using a case study and real world data. It also aims to clearly discuss the approach to determining rigour and validly within thematic analysis as part of a research project. The description of analysis is embedded within the philosophical standpoint of critical realism and pragmatism, which adds depth to the utilisation of these methods in previous discussions [2, 7]. The clear description of the coding and reliability testing used in this analysis will assist replication and will support researchers and doctoral students hoping to demonstrate rigour in similar studies.

This case study was a project investigating the development of effective communication and collaboration tools between acupuncturists and general practitioners (GPs). A GP (sometimes known as a family doctor or family practice physician) is a medical physician whose practice is not orientated to a specific medical speciality but covers a variety of medical problems in patients of all ages [10]. The rationale for this project was the fact that the landscape for patient treatment is changing, and rather than rely solely on their GP’s advice, patients are making their own decisions about choice of treatment. Increasing numbers of patients are using complementary and alternative medicine (CAM) either as an adjunct, or as an alternative to standard mainstream care [11].

There are multiple reasons for the increase in CAM use cited in the literature including dissatisfaction with the biomedical model, increased perceived efficacy of CAM and an increase in training and practice of CAM therapies including biomedical appropriation of CAM skills [12]. Increasingly patients believe a combined approach of CAM and conventional medicine is better than either on its own, and more and more patients have the desire to discuss CAM with well-informed GPs [13].

Patient’s extensive use of CAM has the potential to impact doctor-patient communication. Even with the increase in CAM use and a desire to discuss this, up to 77% of patients do not disclose their CAM use to their general practitioners, and GPs tend to underestimate patients use of CAM and may not ask about it [14]. When CAM is discussed, GPs are being asked about the safety and effectiveness of CAM either on its own or as an adjunct, and additionally many patients have an expectation that their GP will be able to discuss and/or refer to a CAM practitioner [15].

Communication gaps identified in both the research and evidenced in clinical practice formed the basis of this research questions. The specific questions asked were:

1. What is the current communication and collaboration between general practitioners and acupuncturists or CAM?
2. What are the barriers to communication between General Practice and Acupuncture or CAM?
3. How can communication be improved between General Practice and Acupuncture or CAM?
4. Does communication and collaboration differ in the landscape of mental health? And if so, why?

## Methods

The mixed methods project utilised both survey and interview techniques to extrapolate data from the two participant groups (acupuncturists and general practitioners). The research aimed to evaluate and define current practice in order to develop effective strategies to connect the two groups. The tools and strategies allow clinical utility and transferability to other similar clinical groups. Ethics approval for this study was obtained from the The University of Otago Ethics Committee, and additionally the Ngai Tahu Research Consultation Committee approved the research and considered it of importance to Maori health.

The case study example within this article was part of a mixed method project which contained a qualitative approach to interpreting interview data using thematic analysis. It is the analysis of the qualitative component of this study that forms the basis of the discussion contained herewith. These types of qualitative analyses posit that reality consists of people’s subjective experiences or interpretations of the world. Therefore, there is no singular correct pathway to knowledge. This mode of analysis suggests a way to understand meaning or try to make sense out of textual data which may be somewhat unclear. Knowledge is derived from the field through a semi-structured examination of the phenomenon being explored. Thus there is no objective knowledge which is independent of thinking [16].

The case example utilised a codebook as part of the thematic analysis. A codebook is a tool to assist analysis of large qualitative data sets. It defines codes and themes by giving detailed descriptions and restrictions on what can be included within a code, and provides concrete examples of each code. A code is often a word or short phrase that symbolically assigns a summative, salient, essence-capturing, or attribute for a portion of data [17]. The use of a codebook was deemed appropriate to allow for the testing of interpretations of the data, and to allow for demonstration of rigour within the project.

### Study population

The study population consisted of GPs in current practice registered with the Royal New Zealand College of General Practitioners, and Acupuncturists in current practice registered with either the New Zealand Register of Acupuncturists or the New Zealand Acupuncture Standards Authority. Written informed consent was obtained from all participants.

### Sample size

The recommendation for sampling size when investigating the phenomena surrounding experience is six participants [18]. However Guest suggests that thematic saturation is more likely to occur with a sample of twelve [19]. Therefore a group of 27 (14 GPs and 13 acupuncturists) were invited to participate in the semi structured interviews.

Maximum variation purposeful sampling was used for participant recruitment to allow for the exploration of the common and unique manifestations of the target phenomenon and demographically varied cases [20]. Demographic norms were mirrored where possible in the sampling technique with regard to sex, age, ethnicity and type and location of practice. This type of sampling is non-random and is based on the researcher’s viewpoint that the participants selected will provide insightful and penetrating information regarding the research question. Participants initially self-selected by indicating a willingness to be interviewed during the survey phase of this project, and further participants were targeted to meet demographic subsets.

### Rationale of choice of methods

Semi-structured interviews, utilised in this case study, draw on aspects of descriptive research which allow a comprehensive summary of events in everyday terms, and allow for in-depth exploration of a specific phenomenon. The aim is to understand phenomena through meanings that people assign to them [21]. Through the ability to investigate this descriptive data, new perspectives, concepts and themes may be uncovered. During analysis researchers stay close to the data and there is no ability to prove causal effects. Although the inquiry may be value-bound, the researcher aims through the adoption of their ontological and epistemological lens to identify a range of beliefs without introducing bias from their own world view.

These descriptive techniques explored the range of attitudes, perceptions, beliefs and behaviours from the sample and ensured subsequent discussions and proposed interventions were applicable and appropriate to both GPs and acupuncturists. The qualitative data obtained from the semi structured interviews refined and explained the numerical and statistical results from earlier components of the study through a more in-depth exploration.

## Developing the codebook and stages of data coding and testing

The development, use and testing of codebooks is not often reported in qualitative research reports, and rarely in enough detail for replication of the process. The decision to use and test a codebook was important in the demonstration of rigour in this project, as it allowed a clear trail of evidence for the validity of the study and also allowed ease of inter-rater reliability testing of the data. The combination of the inductive/deductive approach described earlier to codebook development meant that the codebook, in this instance, was deduced a priori from the initial search of the literature, the quantitative survey and the initial read of the raw interview data. The preliminary codebook underwent many iterations through the inductive process before the final version was agreed upon by the researchers. (The process of the codebook development is represented in Fig. 1). The utilisation of a codebook allowed a more refined, focused and efficient analysis of the raw data in subsequent reads [8]. The testing of reliability of codes was complex in the context of qualitative research as it could be seen as borrowing a concept from quantitative research and applying it to qualitative research. Yet when adopting the critical realist lens, it is acknowledged that interpretation would be difficult to infer to a wider group without establishing some line of reliability between testers. As this project was aimed directly at practical utility of its findings, the testing approach seemed appropriate.

## Results

### Summarising the data and identifying initial themes

Following the literature review and suggestion of themes for inclusion in the early codebook a priori, the first read of a sample of the raw data was undertaken. The first read of the data involved highlighting text or ‘codable’ units that the researcher considered may become a ‘codable’ moment. Comments were inserted into the margins with initial thoughts and ideas.

This was then done again using the literature informed codes as a guide to determine whether more codable units fitted within the early codebook, or whether further codes needed to be added to the analytical framework.

Examples of each theme, subtheme and code continued to be reviewed and moved until agreement between the coders as to what determined sufficient demonstration of a true representation of a theme became evident. This involved reading and re reading the subset of transcripts multiple times until theme saturation was achieved. Reoccurring themes were identified, but not necessarily given credence over stand out single comments that really embodied a theme.

Codes were written following the guidelines of Boyatzis [9] and were classified with the following: label, definition, description, qualifications or exclusions and examples from the raw data. An example of code labels from this research is outlined in Table 1 below.

**Table 1:** Example of a code definition form the code book

Code Label	Definition	Description	Qualifications or exclusions	Examples
Fear of rejection	Demonstrating anxiety about being cut-off demeaned or isolated. Fear of experiencing hurt, pain or embarrassment due to others’ actions or words	Perceptions of each other based on beliefs or self-held doubts, unfounded opinions, rushing to an opinion without reason.	Can be fear of patient or practitioner being ridiculed - with or without basis	*It’s like some shame based thing. The fear… they fear rejection from the practitioner…. I’ve had plenty of clients who have not told their GP you know, as if they’re having this side relationship with another modality.* ACU013
Feelings of inferiority	Expressing a sense of division within a group of people.	Mention of power imbalance, being treated/acting differently, not feeling the ‘same’, differing world views	Expressed as feelings between clinicians rather than between patients.	*But I don’t tend to do that with GPs, isn’t that interesting? I hadn’t actually thought about that. But I kind of feel like I know the physios better. We seem to be more… more on a level perhaps?* ACU012

### Applying a template of codes and additional coding

Once the codebook was in a draft form it was applied to a larger data set. This was repeated in an iterative way using the early codebook as a guide to determine whether most codable units fitted within the code guide or whether further codes needed to be added to the analytical framework. Once this was done multiple times with no new codes emerging the codebook was assumed as a valid representation of the data.

### Utilising technology: connecting the codes and identifying themes

At this stage the raw data was then transferred into Nvivo software program [22] to allow for a systematic coding approach with the identified codes being added as nodes, and the coded text being matched to the nodes in a systematic way. This allowed for sorting, clustering and comparison of codes between and within subgroups.

### Corroborating and legitimating coded themes

Approaches used for codebook structure analysis were *chunking* and *displaying* [8]. Chunking refers to examining chunks of text that are interrelated and are used for analysis in relation to the research questions and hypotheses. Displaying data is another technique for discovering connections using maps and matrices to contextualise relationships between categories and concepts. Both these approaches allowed for legitimising analysis and to assist in the process of interpretation.

### Testing the reliability of the code

Although inter-rater code testing and discussion occurred throughout the codebook development stage, the final codebook continued to be tested for inter-rater reliability before the data reached the interpretation stage. Reliability can be described as the consistency of judgement that protects against or lessens the contamination of projection [9]. Reliability was tested in this project in two ways:

1. Consistency of judgment over absence and presence (test-retest reliability); and
2. Consistency of judgement across various viewers (inter-rater reliability).

Both tests were checked for reliability using this formula suggested by Miles and Huberman [23]:

$$ \mathrm{Reliability}=\mathrm{number}\ \mathrm{of}\ \mathrm{agreements}/\mathrm{number}\ \mathrm{of}\ \mathrm{agreements}+\mathrm{disagreements} $$

This calculation is a much cruder tool than Choen’s kappa, but gives a simple measure of agreement as a percentage value and is able to be applied to small data sets with a high number of variables. As a rule of thumb, the minimum percentage to demonstrate adequate levels of agreement is 75% [17]. Less than this indicates an inadequate level of agreement.

Inter-rater reliability was tested initially using nominal comparisons of absence or presence of a set of themes and frequency of observation of a single theme (see Table 2). This detailed approach to testing of agreement between coders is not always carried out and/or reported whereas it is suggested here as a necessary step. This continued to be tested, with disagreements being recorded, but minimal changes to the codebook were made at this stage unless it was deemed absolutely necessary as the codebook was now deemed to be in its final version. This was in keeping with the critical realism philosophy that states that various realities are possible, and the pragmatic approach which states that there is no one correct way to code a data set. When all coders were in agreement, these sections of data were determined to be key representations of the code. Where more than one, but not a unanimous agreement was made, this section of data would be discussed between coders. When only one coder applied a section of data to a specific code, this was assumed not to be a clear representation of the code.

**Table 2:** Example of table used for absence/presence reliability

Subtheme/Code	Description					
The role of research.	Mention of how research might inform decisions. Does research impact on relationships with each other? Purpose of research in practice.					
Demonstrating competency	Discussion of what would display competence, what would enable development of trust between practitioners.					
Science vs philosophy when defining acupuncture	Discussion of differing styles of acupuncture training and/or practice. Mention of either specifics of the western or eastern framework.					
Subtheme/code	Coder 1 KR		Coder 2 TD		Coder 3 JN	
	Absent	Present	Absent	Present	Absent	Present
The role of research		Yes		Yes See Table 3		Yes See Table 3
Demonstrating competency		Yes		Yes – first Line 67		Yes p.3 lines 121–127; p. 6–7 lines 300–329
Science vs philosophy when defining acupuncture		Yes		Yes - Line 50, 78, 288		Yes p. 6 lines 287–294

Reliability = 6/6 + 0 = 1 High agreement

Testing Absence/presence of multiple sub-themes/codes within a single interview *Notes for coders- This is a reliability test for coding. Within these single interviews, please record whether the following codes/nodes are absent or present in the interview*

Examples of how reliability testing was undertaken can be found in Tables 2 and 3. Once inter-rater testing had been analysed the coding of the data set was seen as complete and key themes and codes were identified and formed the basis for the discussion in relation to the research questions posed. While absolute agreement was not reached, discussion of coding differences continued to inform the codebook development until all coders agreed that the results were reflective of the data. Of interest here, is that even with this detailed and reflexive process undertaken, the final inter-rater calculations still fell below adequate rates of agreement. However, this clear transparency of the process informed the interpretations and from this, conclusions and recommendations were drawn in relation to the research.

**Table 3:** Example of table used for inter-rater reliability

Participant	Coder 1 KR	Coder 2 TD	Coder 3 JN
ACU001	5 Lines 60–64 259–264 333–342 355–362 371–375	7 Lines 61–63 263/4 322–4 333–339 344–347 355–359 386–388	4 Lines 61–65 259–264 333–350 355–375
GP003	9 Lines 26–29 79–81 100–106 112–115 147–149 243–245 287–294 345–348 362–366	6 Lines 26–29 79–81 100–106 243–245 285–287 345–348	2 Lines 100–108 279–283

Reliability = 23/23 + 10 = .7 – Inadequate agreement reached

Testing frequency of observation of theme ‘research versus relationships’ – subtheme/code ‘the role of research’ in a subset of two interviews *Notes for coders - This is a reliability test for coding to assist with the demonstration of rigour within the data analysis. Within these two interviews, please record the number of times you would code text at ‘the role of research’ node. This relates to the category of defining current practice/research* versus *relationships/the role of research. Coding guide - Mention of how research might inform decisions. Does research impact on relationships with each other? Purpose of research in practice*

## Discussion

### Limitations of this method

A key limitation of codebook development is the extensive time required to establish the codebook itself and to train coders in the use of the codebook. Utilisation of a codebook often requires many revisions and iterations during the code development process before coder agreement can be reached. Even then, and as demonstrated by this project, full inter-coder reliability is unlikely to be achieved. Additionally, application of statistical testing, such as kappa coefficients, would require large volumes of data and analysis is unlikely to be undertaken within most qualitative projects. As was the case with this project, as the code number increases the percentage of agreement decreases during calculation. It is therefore unclear whether this form of analysis would reach a different end point to that of other forms of content analysis. To effectively establish this, a comparison of analysis of the same data using different techniques is recommended for future research projects. However, clear guidelines of qualitative methodological processes will strengthen interpretability and applicability of results.

There are also considerable limitations to the utilisation of the percentage agreement calculation of coders. Frequently one category or code is clearer than others. Thus there is likely to be considerable agreement between the two coders about data in this category. Another problem is that the procedure does not take into account that they are expected to agree solely by chance. A correction for this problem is to use a Kappa statistic to measure the agreement between coders. However, as previously mentioned, the more variables and the larger the data set the more problematic this calculation becomes.

Projection is another limitation of this thematic analysis approach. The stronger a researcher’s ideology the more tempted they will be to project. Projection can be reduced through the development of an explicit code and enabling consistency of judgement through inter-rater reliability. It is also necessary to remain close to the raw information during the development of themes and codes [9]. However, due to the likelihood of different ideologies of coders, it is likely that this will have an impact of the ability to achieve adequate reliability.

Another limitation in thematic analysis can be sampling and the limited scope provided through the use of convenience sampling. In this study the random sample accessed for the quantitative survey did not yield enough voluntary participants for the in-depth interviews hence the sampling framework was modified to purposive convenience sampling. Demographic data was collected to allow for comparison to the larger sample of survey participants and also to the census and workforce survey data carried out nationwide [24, 25]. These demographic comparisons need to be explicitly transparent. In this type of study, the sampling will need to always be a convenience sample. This is because a true random sample is unlikely to be feasible as it is necessary for the interviewee to be willing to participate and this usually involves an interest in the subject or in research itself. This may give a somewhat skewed view within the results which must be taken into account in the analysis.

## Conclusions

This article has provided a detailed and honest account of the difficulties in demonstrating rigour in thematic analysis of qualitative data. However, the researchers involved in this project found the development and utilisation of a codebook, and the application by a team of researchers of the codebook to the data to be advantageous albeit time consuming. It was thought that the codebook improved the potential for inter-coder agreement and reliability testing and ensured an accurate description of analyses. The approach was consistent with the ontological and epistemological framework which informed the study and allowed the unique perspectives highlighted whilst maintaining integrity of analysis. Whilst the creation of the codebook was time intensive, there is the assurance of a demonstration of rigour and reliability within the process. This article has outlined the steps involved in the process of thematic analysis used in this project and helps to describe the rigour demonstrated within the qualitative component of this mixed methods study. This article does not attempt to present an analysis of the data in relation to the research question, but rather provides a clear description of the process undertaken in a qualitative data analysis framework. This article explains and explores the use of Fereday and Muir-Cochranes’ hybrid approach to analysis which combines deductive and inductive coding whilst embedding it in a differing philosophical standpoint [7]. The clear description of the coding and reliability testing processes used in this analysis can be replicated and will support researchers wishing to demonstrate rigour in similar studies. We recommend that others embarking on qualitative analysis as a team embrace and enhance the concept of codebook development to guide complex analytical processes.

## Abbreviations

CAM: Complementary and alternative medicine
GP: General practitioner
